# Supplementary material for: Development of an oral once-weekly drug delivery system for HIV antiretroviral therapy
Source: Nat Commun. 2018 Jan 9;9:2. doi: 10.1038/s41467-017-02294-6 (PMC5760734; doi:10.1038/s41467-017-02294-6)
Supplement: Supplementary file 1 — Supplementary Information [file 41467_2017_2294_MOESM1_ESM.pdf]

# Supplementary Information

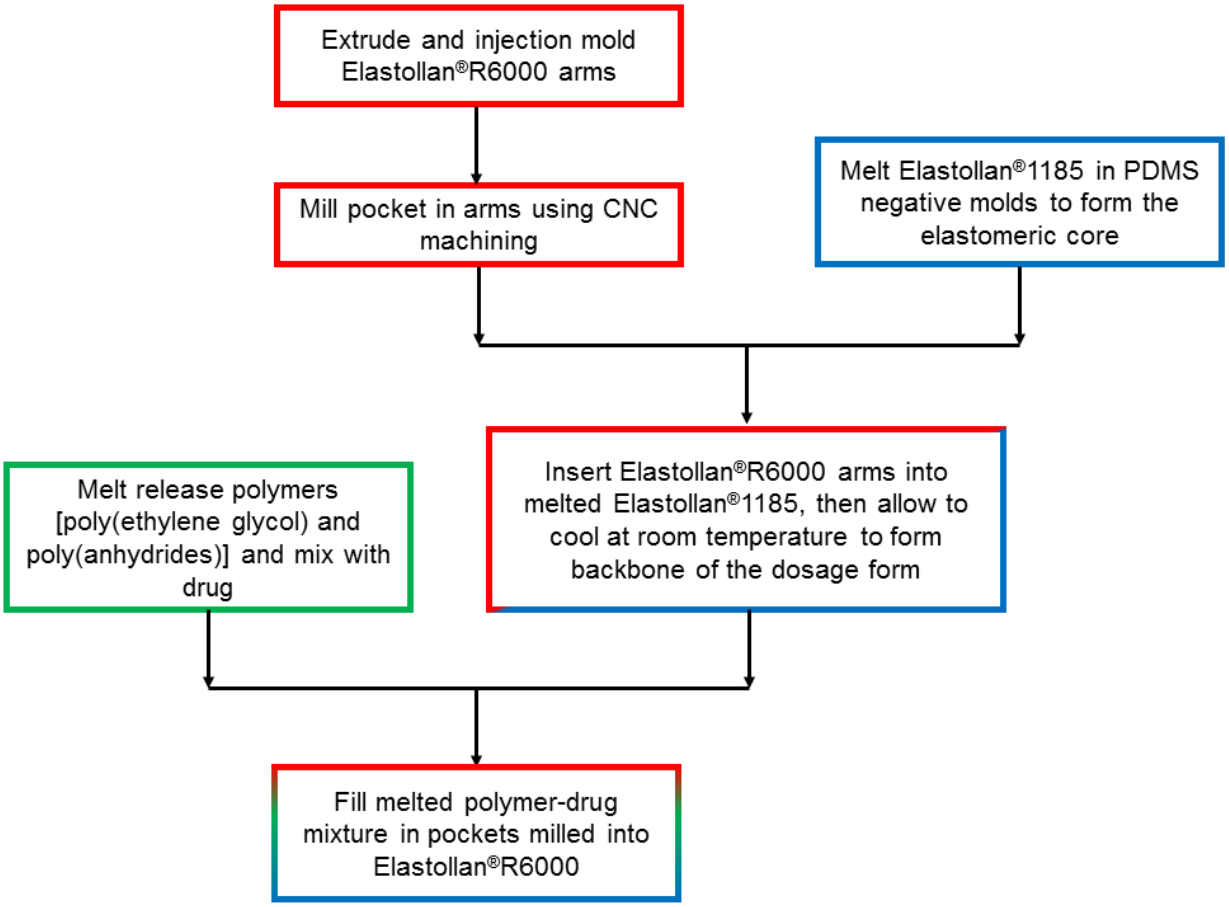


**Supplementary Figure 1: Manufacture of gastric resident dosage forms**

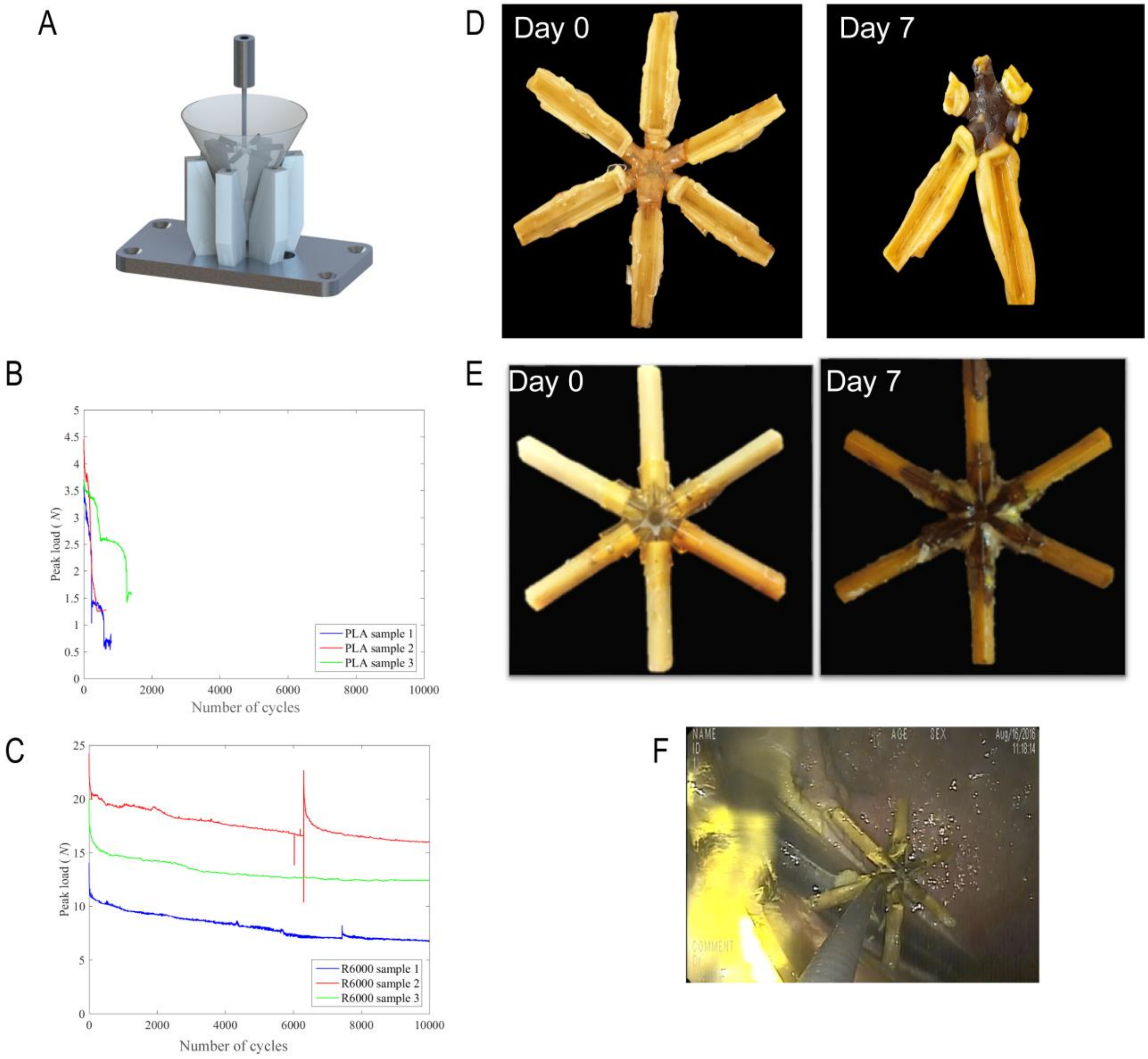


**Supplementary Figure 2: Fatigue testing of dosage forms and images before dosing, during retrieval and after retrieval**

**(A)** Image of set up used for fatigue testing of dosage forms. The dosage forms are pushed by an aluminium rod in a mechanical tester (Instron). Fatigue testing was performed on dosage forms consisting of Elastollan®1185 elastomeric core and (**B**) PLA arms and (**C**) Elastollan® R6000. Dosage forms consisting of PLA arms failed in ~2000 cycles while those consisting of Elastollan®R6000 arms showed no failure for up to 10000 cycles. **(D)** illustrates the dosage form consisting of Elastollan®1185 central elastomer and arms made predominantly of PLA before dosing to pigs (Day 0) and after retrieval from pig stomach (Day 7) **(E)** shows the dosage form consisting of Elastollan®1185 central elastomer and Elastollan®R6000 arms before dosing to pigs (Day 0) and after retrieval from pig stomach (Day 7) **(F)** is an image of the Elastollan®R6000-Elastollan®1185 structure in the pig stomach during retrieval on Day 7**.**

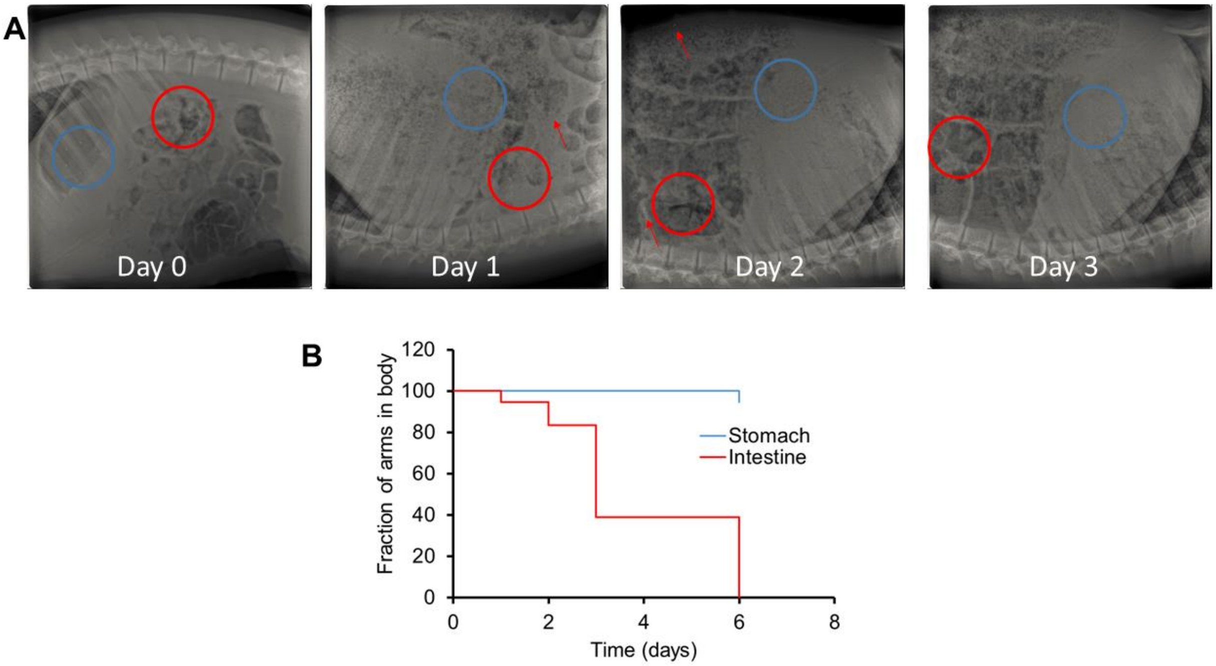


**Supplementary Figure 3: Gastric and intestinal residence of dosage forms**
Dosage forms equipped with an enteric linker were deployed in the stomach and intestine of the same pig. Using serial X-rays we determined the intactness of the dosage form. **(A)** shows representative X-rays of dosage form deployed in the stomach (blue circle) and that deployed in the intestine (red). The red arrow indicates an arm detached from the structure deployed in the intestine. **(B)** indicates the survival of the arms deployed in the stomach and intestine. Three structures were deployed in the stomach and three in the intestine with each consisting of 6 arms.

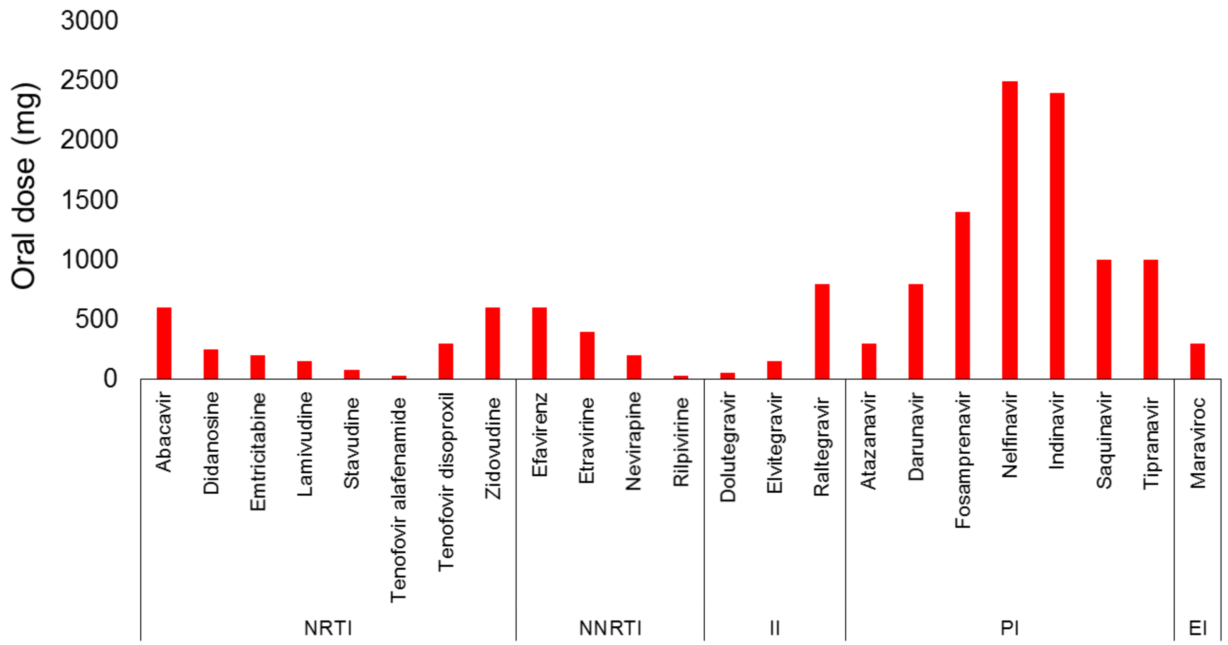


**Supplementary Figure 4: Approximate recommended daily doses of FDA approved antiretrovirals**

Figure shows the approximate daily dose of FDA approved antiretrovirals. DTG, RPV and TAF have daily doses of ≤50 mg. NRTI: Nucleoside reverse-transcriptase inhibitor; NNRTI: Non-nucleoside reverse-transcriptase inhibitor; II: Integrase inhibitor; PI: Protease inhibitor; EI: Entry inhibitor. CAB was used in these studies, but is not FDA approved yet and hence not included in this chart. In clinical studies, CAB has been used at a daily dose of ~30 mg.

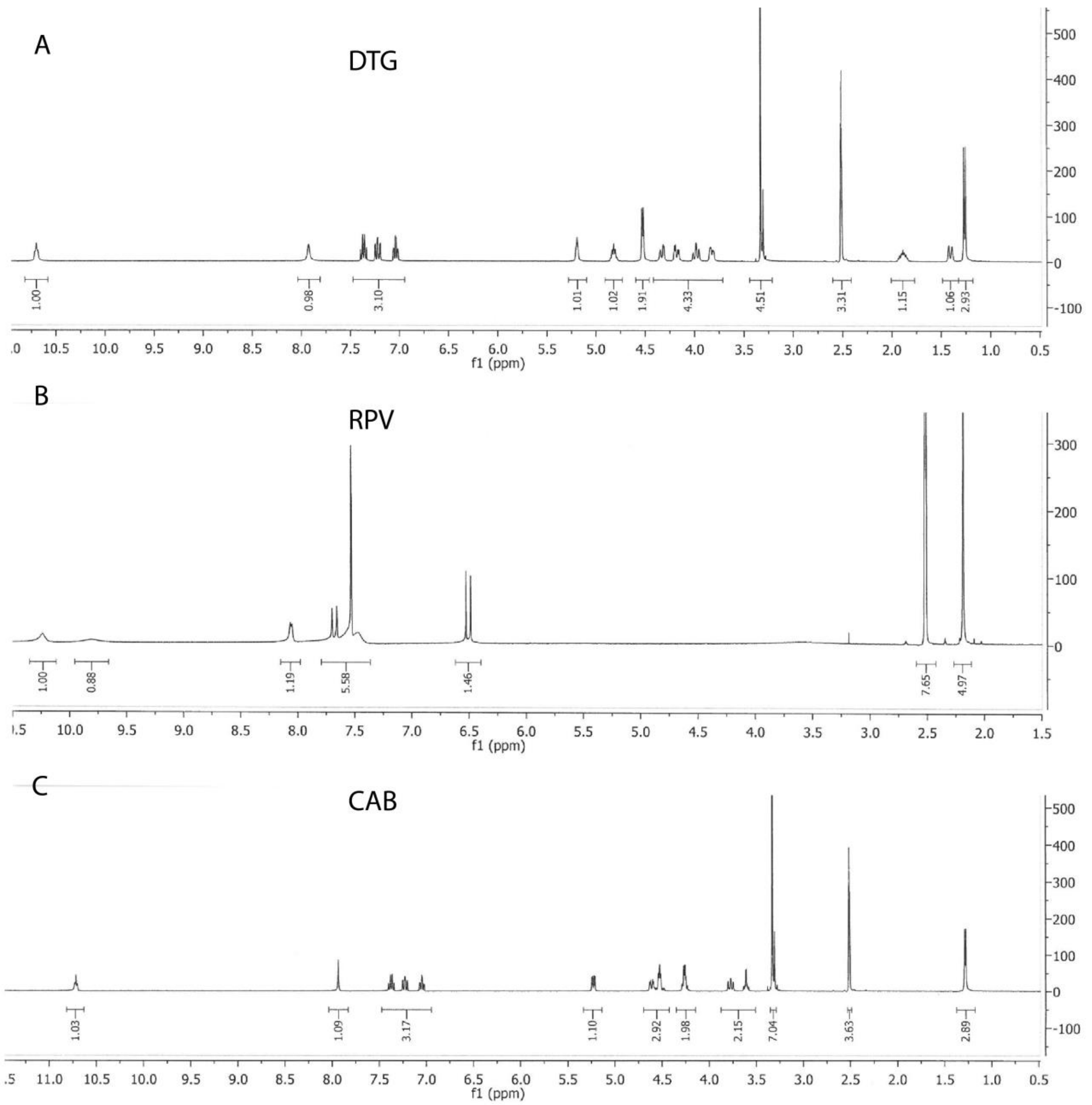


**Supplementary Figure 5: NMR analysis of antiretroviral drugs**
(A) DTG (B) RPV and (C) CAB were dissolved in DMSO-d6 and analysed using NMR

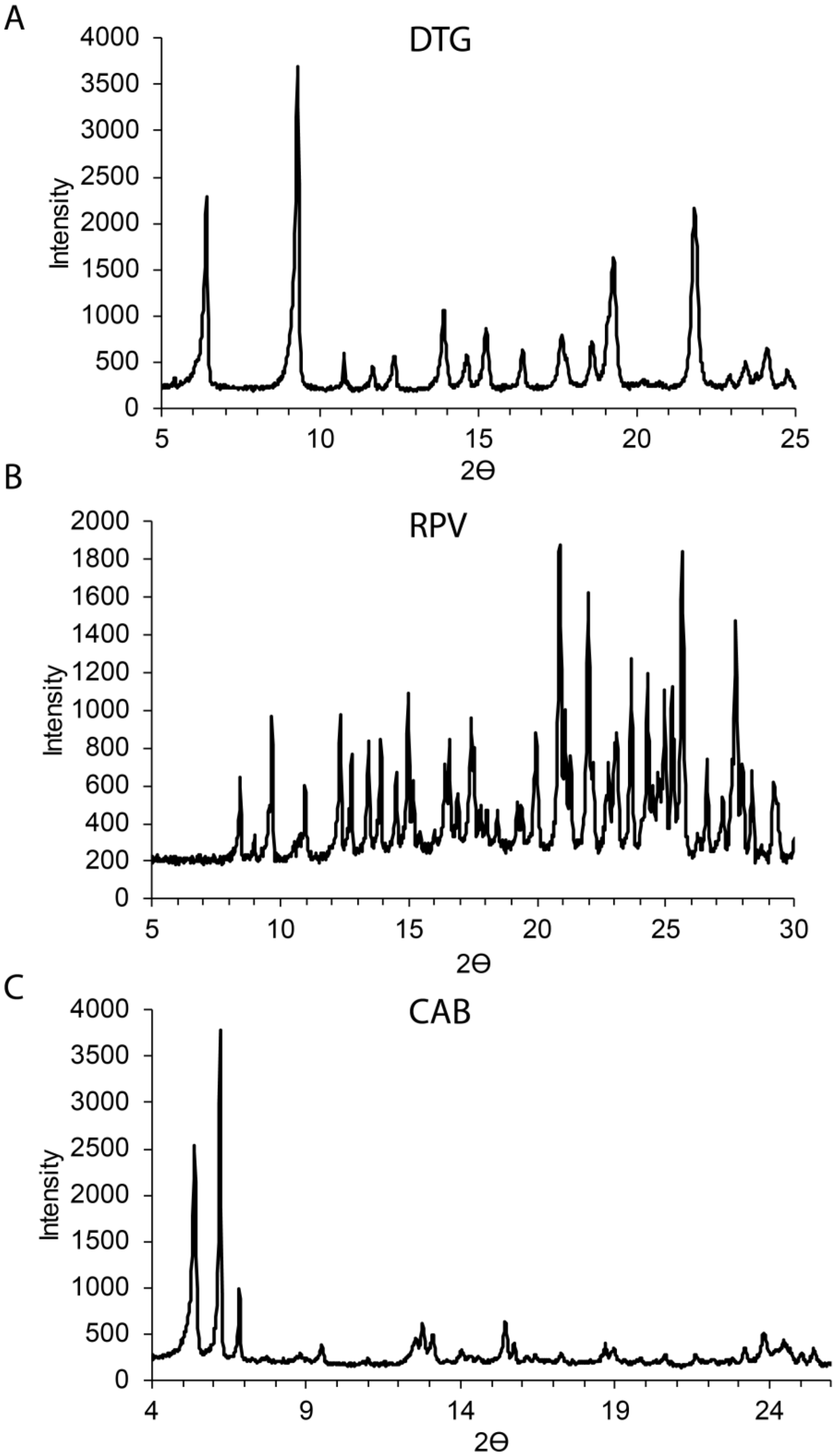


**Supplementary Figure 6: X-ray diffraction analyses of antiretrovirals**

Shown here are the XRD patterns of (A) DTG (B) RPV and (C) CAB

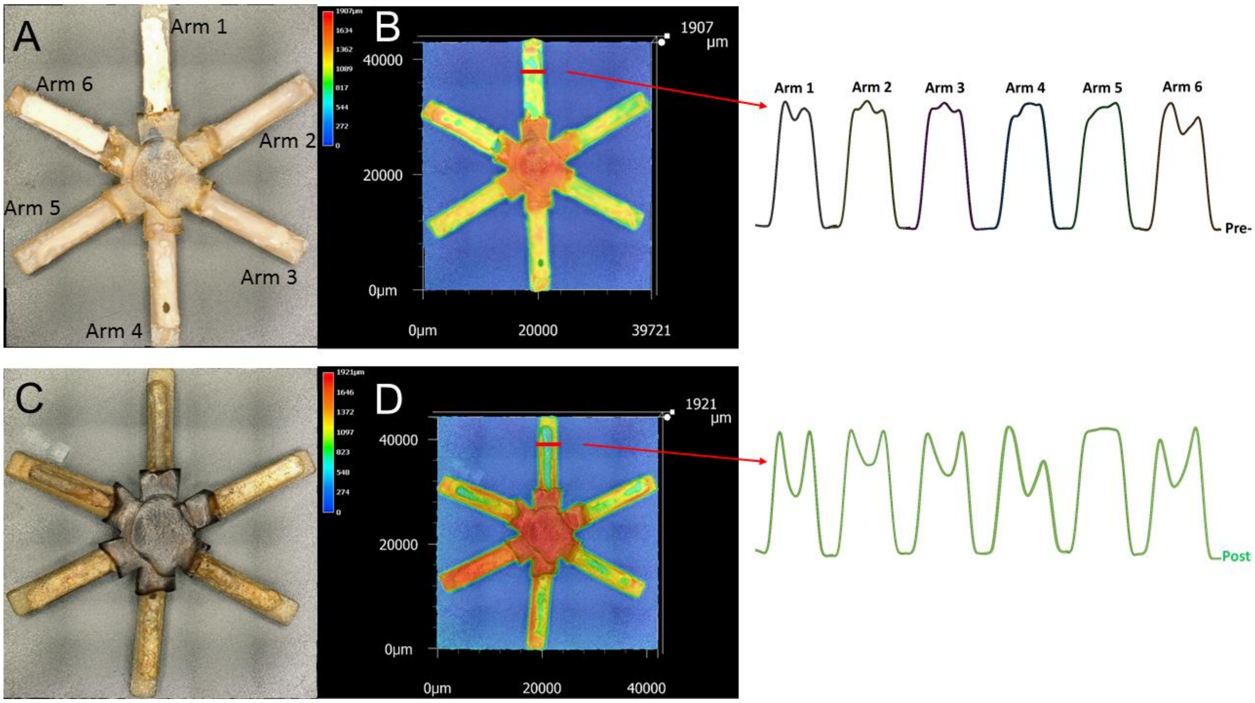


**Supplementary Figure 7: Microscopic analysis of DTG dosage forms before dosing and after retrieval**

**(A)** is an image of the dosage form before dosing. **(B)** shows the heat map indicative of the height of material at each position along with the line profile indicating the level of material along the breadth (shown by red line) of each arm. **(C)** is an image of the dosage form after retrieval from the pig 7 days after dosing. **(D)** shows the heat map of level of material remaining in the dosage form with line profiles indicating the level of material along the breadth of each arm. Arm 5 contained one of the slowest releasing polymers, and did not show significant loss of structure.

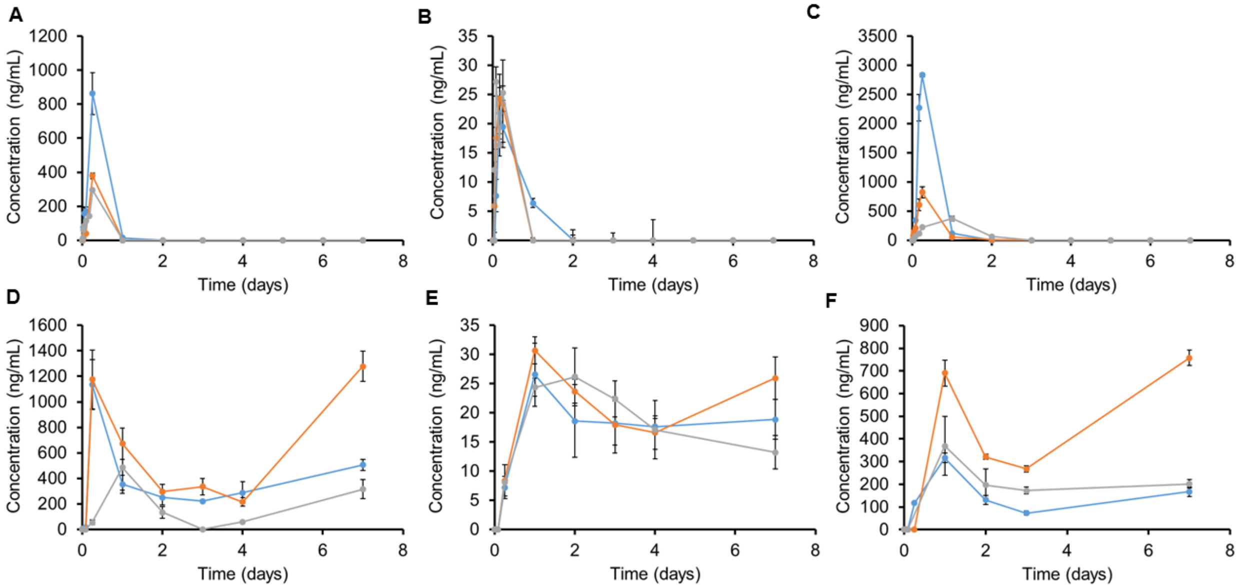


**Supplementary Figure 8: Concentration-time profiles of ARVs in individual animals**
Pharmacokinetics of immediate release formulations of **(A)** DTG **(B)** RPV and **(C)** CAB and sustained release formulations of **(D)** DTG **(E)** RPV and **(F)** CAB. Data is represented as mean ± S.D., n=3.

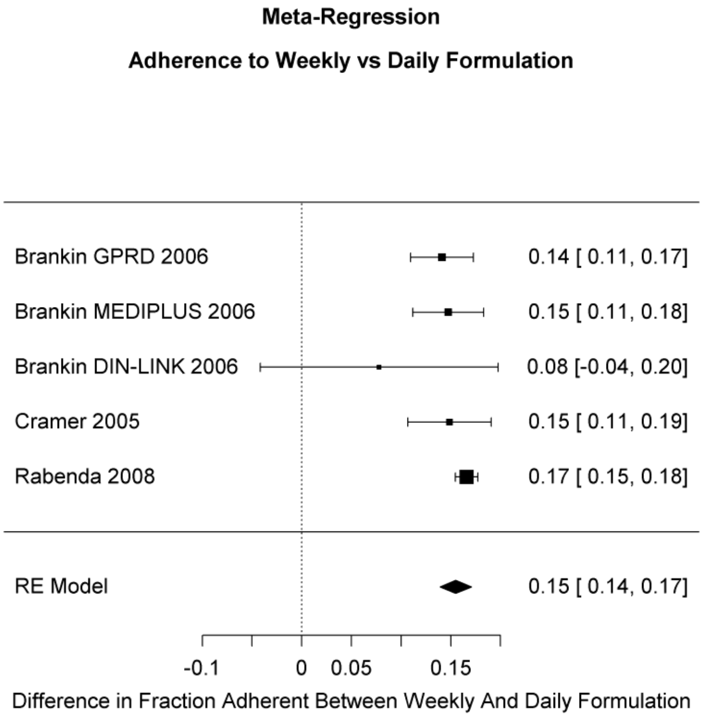


**Supplementary Figure 9: Meta-regression of adherence increase by switching to weekly formulation**

The random-effects (RE) model accounting for both study effect size and between-study variance estimates 15% (95% CI: 14-17%) increase in fraction adherent (i.e. MPR or PDR above 80%) when switching from daily to weekly formulation.

**Supplementary Table 1: Formulations of various drugs used for the pharmacokinetic studies**

| Drug \ Polymer | Poly(ethylene glycol) + Kollidon VA64 | Poly(adipic anhydride) | Poly(adipic anhydride) + Poly(sebacic anhydride) | Poly(sebacic anhydride) |
|---|---|---|---|---|
| **Dolutegravir** | 1/6 | 1/2 | 1/6 | 1/6 |
| **Rilpivirine** | 1/12 | 3/4 | 0 | 1/6 |
| **Cabotegravir** | 1/6 | 1/2 | 0 | 1/3 |

**Supplementary Table 2 Scaling factors relating concentrations of $CD4^+$ T cells and virus in blood to total body levels.** In calculating total plasma volume, a weight of 70 kg is assumed.

| Parameter | Description | Value | Source |
|---|---|---|---|
| $V_{plasma}$ | Total body plasma volume | 3 L | [69] |
| $f_{bp}$ | Fraction of blood that is plasma | 0.6 | [69] |
| $f_{cb}$ | Fraction of total body CD4 cells in peripheral blood | 0.02 | [70] |
| $\delta_{CD4}$ | Total CD4 cells : CD4 count | $2.5\times10^8$ μL blood | $10^6\ V_{plasma}/f_{bp}f_{cb}$ |
| $x_0$ | Baseline CD4 count | 1000 cells/μL blood | |
| $\delta_v$ | Total virions: viral load | $7.5\times10^4$ / RNA/mL plasma | $500\ V_{plasma}/f_{cb}$ |

**Supplementary Table 3: Viral dynamic parameters for wild-type strain in the absence of drug therapy.** Values were estimated based on sources cited.

| Parameter | Description | Value | Source |
|---|---|---|---|
| $R_0$ | Basic reproductive ratio | 10 | [21,71] |
| $\lambda$ | Production rate of target cells | $1.35\times10^{10}$ cells/day | $\lambda = \delta_{CD4}\, x_0\, d_x$ |
| $d_x$ | Death rate of target cells | 0.05/day | [72] |
| $\alpha$ | Fraction of cells supporting productive infection | 0.01 | [32–34] |
| $d_y$ | Death rate of infected cells | 1/day | [73] |
| $k$ | Viral burst size | 2500 virions/cell | [74] |
| $c$ | Clearance rate of free virus | 25/day | [75] |
| $\beta$ | Infectivity rate of virus | $1.11\times10^{-3}$ /cell/day | $\beta = R_0\, d_y\, d_x\, c/\, \alpha\, \lambda\, k$ |
| $A$ | Exit rate of latent cells | 100/day | [66] |

**Supplementary Table 4: Drug parameters used to estimate drug efficacy.** $C_{max}$: Maximum plasma concentration achieved shortly after dosing, at steady state, with perfect adherence. Half-life ($\tau_{1/2}$): time for drug levels to decay to half of initial values. $IC_{50}$: Drug level at which viral infectivity is reduced to 50% of the drug-free value. $m$: slope parameter for a sigmoidal Hill-curve describing the concentration response of the drug. Pharmacodynamic values ($C_{max}$ ,$m$) are from the Siliciano laboratory [46,55–57], and are based on a single-round infectivity assay using a GFP-encoding, envelope-deficient version of the standard HIV reference strain NL4-3. Pharmacokinetic parameters are from the FDA label [58].

| Drug | $C_{max}$ (μM) | Half-life, $\tau_{1/2}$ (hrs) | $IC_{50}$ (μM) | $m$ | Molecular weight (g/mol) |
|---|---|---|---|---|---|
| DTG | 8.75 | 14 | 0.07 | 1.2 | 419 |
| RPV | 0.51 | 50 | 0.105 | 1.83 | 366 |

**Supplementary Table 5**: **Nucleotide frequency and mutation matrix for HIV genome**. The center portion of the table gives the probability per site, per round of viral replication, of a substitution between the original and mutated nucleotide. Details of the calculation of this matrix are given in [21], and used data from intracellular *in vitro* experiments [60,61]. The final column of the table gives the frequencies at which each of the nucleotides occurs in the overall HIV genome [62].

| | | Mutated nucleotide | | | | Frequency |
|---|---|---|---|---|---|---|
| | | U | C | A | G | |
| Original nucleotide | U | | $1.1\times10^{-5}$ | $1.4\times10^{-5}$ | $3.7\times10^{-6}$ | 0.223 |
| | C | $2.5\times10^{-5}$ | | $6.7\times10^{-6}$ | $1.8\times10^{-7}$ | 0.182 |
| | A | $8.2\times10^{-7}$ | $5.5\times10^{-7}$ | | $1.2\times10^{-5}$ | 0.351 |
| | G | $8.8\times10^{-7}$ | $8.8\times10^{-7}$ | $5.7\times10^{-5}$ | | 0.244 |

**Supplementary Table 6: Resistance mutation parameters for dolutegravir (DTG).** Empty cells represent missing values, for which we assumed $\sigma = 0$ and $s = 0.05$.

| Mutation | Fold change in $IC_{50}$ ($\rho$) | Source | Fractional change in slope ($\sigma$) | Source | Cost of resistance ($s$) | Source |
|---|---|---|---|---|---|---|
| M50I | 1.94 | 76 | | | 0.08 | 76 |
| H51Y | 1.25 | 76 | | | 0.11 | 76 |
| E138K | 0.40 | 76 | | | 0.36 | 77 |
| G140S | 0.76 | 57 | -0.01 | 57 | 0.47 | 57 |
| Q148K | 1.10 | 78 | | | 0.89 | 78 |
| Q148R | 1.20 | 78 | | | 0.64 | 78 |
| N155H | 1.96 | 57 | | | 0.35 | 57 |
| E157Q | 0.01 | 79 | | | 0.19 | 79 |
| R263K | 2.00 | 76 | | | 0.30 | 76 |
| M50I/R263K | 15.59 | 76 | | | 0.30 | 76 |
| H51Y/R263K | 6.95 | 76 | | | 0.89 | 76 |
| E138K/Q148K | 19.00 | 78 | | | 0.81 | 78 |
| G140S/Q148R | 7.98 | 57 | 0.01 | 57 | 0.57 | 57 |
| Q148R/N155H | 10.00 | 80 | | | | |
| E157Q/R263K | 19.90 | 79 | | | 0.00 | 79 |

**Supplementary Table 7: Resistance mutation parameters for rilpivirine (RPV).** Empty cells represent missing values, for which we assumed $\sigma = 0$ and $s = 0.05$.

| Mutation | Fold change in $IC_{50}$ ($\rho$) | Source | Fractional change in slope ($\sigma$) | Source | Cost of resistance ($s$) | Source |
|---|---|---|---|---|---|---|
| K101P | 102.13 | 56 | -0.21 | 56 | 0.70 | 56 |
| K101E | 3.00 | 81 | | | 0.80 | 82 |
| K103N | 1.23 | 56 | 0.12 | 56 | 0.30 | 56 |
| E138K | 2.30 | 81 | | | 0.66 | 83 |
| Y181I | 152.91 | 56 | -0.10 | 56 | 0.44 | 56 |
| Y181C | 1.72 | 56 | -0.48 | 56 | 0.26 | 56 |
| Y181V | 31.00 | 84 | | | | |
| M184I | 1.50 | 85 | | | 0.66 | 83 |
| M184V | 0.80 | 85 | | | 0.46 | 56 |
| Y188L | 6.00 | 86 | | | | |
| M230L | 3.40 | 87 | | | | |
| K101E/E138K | 3.20 | 81 | | | 0.00 | 81 |
| K101E/M184I | 5.80 | 85 | | | | |
| K101E/M184V | 2.20 | 85 | | | | |
| Y181C/Y188L | 30.20 | 87 | | | | |
| E138K/M184I | 3.20 | 81 | | | 0.00 | 83 |
| E138K/M184V | 3.10 | 85 | | | 0.00 | 83 |
| E138K/M230L | 22.60 | 87 | | | | |

**Supplementary Note 1**
**SUMMARY**

To predict how individual patients respond to long-acting drug formulations, we adapted an established model of within-host HIV dynamics in the presence of antiretroviral therapy [21]. The backbone of this model tracks the virus and its target cells throughout the plasma and lymph tissue and is calibrated to clinical data. We model the impact of treatment by allowing viral infectivity to depend on drug levels, which fluctuate over time (termed pharmacokinetics), and drug efficacy, determined by the $IC_{50}$ and slope of the dose-response curve of the particular drug (pharmacodynamics). Both drug-sensitive and drug-resistant viral strains are tracked and interact via mutation and selection. Our model is similar to those that have been used previously to predict the dynamics of HIV[22–24], hepatitis B virus [25], hepatitis C virus [26,27], herpes simplex virus 2 [28], and various bacterial infections [29,30].

## MODEL

### Viral dynamics model

We use a slightly modified version of the well-established viral dynamics model to describe the dynamics of multiple strains of HIV within an individual patient [21,31]. This model tracks uninfected target cells of the virus ($CD4^+$ T cell , $x$), actively infected cells ($y$), and free virus ($v$). Described as a system of differential equations, the model is

$$\begin{aligned}\dot{x} &= \lambda - \sum_{i=1}^{n} \beta_i x v_i - d_x x \\ \dot{y}_i &= A_i + \alpha \sum_{j=1}^{n} Q_{ji} \beta_j x v_j - d_y y_i \\ \dot{v}_i &= k_i y_i - c v_i\end{aligned} \tag{1}$$

Uninfected target cells are produced at a rate $\lambda$ and die at a rate $d_x$. Infection occurs at a rate $\beta_i$ per contact between uninfected cell and free virus of strain $i$. Mutations can occur during the reverse-transcription phase, and so infection with virus of strain $i$ can lead to an infected cell with strain $j$ with rate $Q_{ij}$, where $\mathbf{Q}$ is the mutation rate matrix [21,31]. Here we will track wildtype drug-susceptible virus, a suite of drug-resistant strains, and intermediate genotypes along the pathway to resistance. To account for the observation that only a small fraction of $CD4^+$ T cell are infected even in advanced infection, and yet levels of these cells are dramatically reduced by infection, we assume that only a fraction $\alpha$ of target cells contacted by the virus will proceed to productive infection, while the remainder will be rapidly killed by the virus (e.g. by pyroptosis or another means of "bystander" cell death)[32–34]. Infected cells produce free virus at rate $k$ and die at rate $d_y$. Free virus is cleared at rate $d_v$. Infected cells can also be produced by reactivation of latent infection at rate $A_i$.

This model reproduces many of the important features of HIV infection. During acute infection, the virus starts at low levels and grows approximately exponentially to a peak level, before declining to a lower "set-point" viral load where it remains approximately constant for many years [35,36]. Total CD4 levels decline substantially during infection, even if only a small fraction of cells are productively infected [37,38]. If viral replication is blocked by effective treatment,

then viral load levels decay until they reach a low level sustained by reactivation of latent infection [39,40]. CD4 T cells eventually recover [39]. If treatment is interrupted at any point, viral levels rapidly rebound [41,42].

The fitness of any particular viral strain can be summarized in a single composite parameter known as the basic reproductive ratio ($R_0$):

$$R_{0i} = \frac{\alpha \lambda \beta_i k_i}{d_x d_y d_v} \tag{2}$$

This value describes the average number of secondary infections caused by a single infected cell over its entire lifespan. If $R_0 > 1$, this strain can undergo continual self-sustaining rounds of replication and reach high levels. When $R_0 < 1$, the strain will be unable to sustain infection, and will only be maintained due to reactivation from latency or production via mutation of another strain. If multiple strains all have $R_{0i} > 1$, the one with the highest value will outcompete the others to reach high levels, and the others will persist only at low levels.

Here we assume, for simplicity, that strains differ only in their infection rates, $\beta$, and their frequency in the latent reservoir, $A$. We assume that the latent reservoir maintains a constant size, instead of explicitly modeling its dynamics. This is a very good approximation when we are modeling treatment initiated after acute infection, when the majority of reservoir seeding occurs, and after which size is relatively constant [43,44].

This model can also be implemented stochastically to properly account for the finite size of infection and rare events such as mutation and reactivation from latency (see below).

## Treatment with antiretroviral therapy

Antiretroviral therapy acts by preventing infection at a particular stage of the viral lifecycle. The action of all current antiretroviral drugs – including fusion inhibitors, reverse transcription inhibitors, integrase inhibitors, and protease inhibitors (which lead to production of non-infectious virus) – can be described by reductions in the infection parameter $\beta$. The efficacy of a drug as a function of the drug concentration (the pharmacodynamics) is well-described empirically by a Hill-type sigmoidal dose-response curve [21,45,46]:

$$\beta(D) = \frac{\beta}{1 + (D/IC_{50})^m} \tag{3}$$

where $\beta$ is the infectivity in the absence of drug, $D$ is the drug concentration, $IC_{50}$ is drug level at which infectivity is reduced by half, and $m$ is the slope parameter which describes the steepness of the curve.

If multiple drugs are given in combination, the efficacy equation can be adjusted to account for the combined effect of all drugs, including any drug interactions [47].

HIV treatment is always taken as discrete doses and consequently drug levels $D(t)$ vary over time. We use a simplified description of these pharmacokinetics, in which drug concentrations instantaneously increases after a dose to a peak concentration and then decay exponentially (with half-life $\tau_{1/2}$) to a trough concentration before the subsequent dose:

$$D(t) = D(0) 2^{-t/\tau_{1/2}} \tag{4}$$

where $t$ is the time since the last dose.

When a drug is taken continuously with the prescribed dose interval $\Delta t_d$, it reaches a steady-state where the peak level is termed $C_{max}$ and the trough level is $C_{min} = C_{max} 2^{-\Delta td/\tau 1/2}$. The difference

between these concentrations, $\Delta C = C_{max} - C_{min}$, describes the amount by which each subsequent dose increases the drug level, even when doses are missed or before steady state is achieved.

We model imperfect adherence to antiretroviral therapy by assuming each prescribed dose is taken randomly and independently, at the prescribed time, with a probability given by the average adherence level ($p_A$).

### Long-acting antiretroviral therapy

To model a hypothetical long-acting formulation of antiretroviral therapy [48–51] we assumed a dosage form existed with identical pharmacodynamics parameters but with a longer half-life and less frequent administration. As a baseline case, we assumed the peak concentration $C_{max}$ was the same, but also explored lower values. Otherwise the parameters for long-acting antiretrovirals were assumed to be the same as for the daily formulation.

For hypothetical weekly long-acting formulations, we considered two different potential adherence scenarios. The first, weekly-choice adherence, is simply a weekly analogue of the case for existing drugs. We assume that the patient has a fixed day they are supposed to take a new pill (e.g. Monday), and each week on this day, they randomly decide, with their adherence probability $p_A$, whether or not they will take the pill for that week. If they do not take it, they must wait until the next week to do so. This scenario may represent a particular behavioral strategy, or could occur if pills are only made available to patients on a weekly basis. We also consider a more optimistic scenario for weekly long-acting therapy adherence, which we call daily-choice adherence. In this situation, patients may decide to take their next dose on any day starting one week after their previous dose. Each day after this point, they (randomly) decide with probability $p_A$ whether or not to start the next week-long dose. In this case, for any value of $p_A$, the chance that this individual will go a whole week without ever deciding to take their next pill is much lower. We think this scenario may be a natural consequence of human decision-making behavior and represents a possible way in which long-acting formulations may effectively increase adherence.

## PARAMETER VALUES

### Viral dynamic parameters

Generally viral dynamics models track infection only in the blood, since that is the compartment most readily sampled during routine clinical care. However, the majority of the infection takes place in the lymph tissues, which has been shown in many studies to be well-mixed with the blood [52,53]. Because the study of resistance requires tracking total body levels of infection, we derived scaling factors to relate the full-body infection size to levels in the blood. To relate the blood concentration of $CD4^+$ T cell ($x$) to the total body level ($X$), we use the following scaling:

$$\begin{aligned} X &= \left(\frac{\text{Blood}}{\text{Plasma}}\right) \times (\text{Total plasma volume}) \times \left(\frac{\text{Total CD4}^+\text{ T cell}}{\text{Periph. blood CD4}^+\text{ T cell}}\right) \\ &\quad \times \left(\frac{10^6\ \mu\text{L}}{1\text{ L}}\right) \times \left(\frac{\text{CD4}^+\text{ T cell}}{\mu\text{L blood}}\right) \\ &= \frac{1}{f_{bp}} \times V_{plasma} \times \frac{1}{f_{cb}} \times 10^6 \times x \\ &= \delta_{CD4} x \end{aligned} \tag{5}$$

where $\delta_{CD4} = 10^6 V_{plasma} / f_{bp} f_{cb}$. Similarly, to scale virus levels measured as viral RNA copies per mL of plasma ($v$) to total body virions ($V$), we assume that virus distributes between the periphery and lymph with the same proportion as cells and use:

$$V = \left(\frac{1 \text{ virion}}{2 \text{ RNA copies}}\right) \times V_{plasma} \times \frac{1}{f_{cb}} \times \left(\frac{10^3 \text{ mL}}{1 \text{ L}}\right) \times v \\ = \delta_v v \tag{6}$$

where $\delta_v = 500 V_{plasma} / f_{cb}$. Supplementary Table 2 gives the values we used for these scaling values and their sources. Supplementary Table 3 gives the parameters used in the viral dynamics model for the wildtype virus. Alterations to viral fitness caused by the drug and resistance mutations are described by the parameters given in the next section.

With these parameter values, pre-treatment total body levels of infected cells and free virus are of order $10^8$ and $10^{10}$, respectively, and healthy levels of $CD4^+$ T cells are of order $10^{11}$ (in agreement with experimental data [54]).

**Pharmacodynamic parameters**

For all drugs, $IC_{50}$ and slope values for wild-type virus were determined by *ex vivo* laboratory experiments conducted in the Siliciano laboratory at Johns Hopkins School of Medicine [46,55–57] (Supplementary Table 4). We consider the methods of this group to be the gold standard, because a) they use a single-round infectivity assay, b) they use a highly quantitative fluorescent-cell-sorting readout, c) they use primary $CD4^+$ T cells and plasma from donors, d) they generate resistance mutations in a constant genetic background. They additionally characterized a suite of resistance strains, which is any viral variant that is less inhibited than the wild type for some drug concentration. Each resistant strain is characterized by three parameters that describe the alterations to the dose response curve of viral infectivity relative to the wild type ($\beta$):

$$\beta' = \frac{\beta(1-s)}{1 + \left(\frac{D}{\rho IC_{50}}\right)^{m(1+\sigma)}} \tag{7}$$

Resistance mutations increase the $IC_{50}$ of the dose response curve by a factor $\rho$ and also in general confer a fitness cost, meaning that in the absence of the drug, infectivity is reduced compared to the wildtype ($0 < s < 1$). This fitness cost is often alternatively described by the replication capacity, $1 - s$. Many mutations also reduce the slope ($m$) of the dose-response curve by a fraction $\sigma < 0$ [55].

We also considered other sources of data on resistant strains that were not characterized by the Siliciano group. These other sources do not always meet all the assay standards outlined above, and often only measure the fold-change in $IC_{50}$ ($\rho$) of resistant strains, omitting the fitness cost ($s$) or changes in the slope ($\sigma$). When these values were missing, we assumed $\sigma = 0$ and $s = 0.05$ (Supplementary Tables 6, 7).

**Pharmacokinetic parameters**

For existing immediate-release formulations, the maximal plasma concentration ($C_{max}$) and the half-life ($\tau_{1/2}$) were taken from the FDA drug labels [58] (Supplementary Table 4). For long-acting formulations, we consider a hypothetical scenario. We believe that the pharmacokinetic profiles observed in the swine model in this preliminary study are likely to be different than those observed in eventual human testing, both because drug absorption and elimination are species-specific and

because the current dosage forms are hand-made in the laboratory but will undergo extensive improvements and industrial-grade manufacturing before administration to humans. Therefore, we did not focus on characterizing the swine pharmacokinetics in detail and exactly reproducing them in our model. Instead, we assumed a relatively simple profile for a hypothetical clinical long-acting formulation. Since the form will be given once a week, we assumed that the half-life was seven-fold longer than the existing formulation. As a baseline case, we assumed the peak concentration $C_{max}$ was the same, but also explored lower values. Otherwise the parameters for long-acting antiretrovirals were assumed to be the same as for the daily formulation.

### Adherence

For some results, we present predicted patient outcome as a function of the input adherence level, where adherence was varied from 0 to 100% in increments of 5%. In other results, we present outcomes for a hypothetical cohort of treated individuals with a distribution of adherence levels. We use an empirically-determined distribution of adherence levels to antiretroviral therapy taken from a study using unannounced pill counts [59].

### Mutation rates

The entry $Q_{i,j}$ in the mutation rate matrix **Q** describes the probability that entry of viral strain *i* into a host cell will lead to infection with strain *j*, which is just the probability that the viral genotype *i* is mutated to produce strain *j* during reverse transcription. Working out this rate for any particular mutation of interest requires multiple steps. Mutations that confer drug resistance to HIV are described by their genotype at the amino acid level: for example, M184V is a mutation in the reverse transcriptase gene in which the naturally-occurring amino acid methionine (M) at position 184 is changed to a valine (V). To determine the rate at which such a mutation occurs, we need to know which triplet of nucleotides code for the M and V amino acids (redundancy in the genetic code means there will be multiple codons for each), at which frequency each possible codon is expected to exist, which nucleotide could be changed to produce the amino acid change, and at what rate each of those nucleotide mutations could occur. To do this, we use the well-known human codon table, the overall nucleotide composition of the HIV genome, and the nucleotide-to-nucleotide mutation matrix for HIV [21,60–62] (Supplementary Table 5). We then apply the following algorithm:

- Break the genotype down into single amino acid changes (e.g. E138K/M184V→E138K,M184V)
- Add each single amino acid change and all possible combinations of them into the mutation matrix as strains
- For each single amino acid change (e.g. E138K)
    - determine all possible codons for the wildtype amino acid (e.g. E = {GAA,GAG})
    - estimate the frequency of occurrence of these wildtype codons based on the known nucleotide composition of the HIV genome (59% GAA, 41% GAG)
    - determine all possible codons for the mutant animo acid (e.g. K = {AAA,AAG})

- find all possible single-nucleotide changes that could turn the wildtype amino acid into the mutant animo acid, and the rate at which these changes would occur (GAA→ AAA, GAG→AAG)
- calculate the total rate of the amino acid change by summing the rate of all possible nucleotide changes, weighted by their estimated frequency of occurrence (rate = $0.59 \times 5.7{\times}10^{-5} + 0.41 \times 5.7{\times}10^{-5} = 5.7{\times}10^{-5}$)
- If there is no possible single-nucleotide change to go from the wildtype to mutant amino acid (e.g. K101P)
    - find all intermediate animo acid states that could be passed through to get from wild- type to mutant animo acid. Add these as strains to the mutation matrix (K101Q, K101T)
    - For each of these intermediates
        - use the same method as above to calculate the rate of single nucleotide changes for each step (wildtype to intermediate, intermediate to mutant)

- For each mutation requiring multiple amino acid changes, find all strains with all but one of these mutations, and enter the rate to get the final genotype as the same rate at which that final genotype alone would occur from the wild type strain (e.g. M184V→E138K/M184V occurs at the same rate as wild type →E138K: $5.7{\times}10^{-5}$)
- For all strains, calculate the probability of this strain directly mutating to or from any other strain. If only a single nucleotide change is needed, we use the method above, and if multiple nucleotide changes are needed, we calculate the rate of each single-nucleotide change and take their product (e.g. wild type →E138K/M184V at rate $5.7{\times}10^{-5} \times 1.15{\times}10^{-5}$)
- For each strain, calculate the probability that a mutation does not occur, by requiring that the sum of the values across a row is 1.

Note that this last step assumes that any mutations not accounted for in the mutation matrix are neutral and equivalent to the wild type, including mutations which may include combinations of the resistance mutations listed that have not yet been observed or phenotyped, and therefore not included in our analysis. In this way, we apply a closure approximation to the mutation matrix instead of modeling a full matrix of $2^n$ possible genotypes that could be constructed out of n possible mutant loci.

These calculations assume that the mutation rate is independent of the genetic background (other than depending on the initial and final nucleotide) and that synonymous mutations are neutral and their relative frequencies are described by the overall base composition of HIV. In the absence of drug treatment, drug-resistant mutants will still be produced by mutation, but will be selected against due to their fitness cost. The balance between these two processes results in their persistence at an average level described by mutation-selection balance [31,63]. This persistence level can be calculated by constructing a new matrix **M** where $M_{i,j} = Q_{i,j}\beta_i$, and then the frequency $f_i$ of each strain $i$ is given by the $i^{th}$ entry in the (normalized) dominant left eigenvector of **M.** We assume that mutant viral strains exist at mutation-selection balance both in actively infected cells at the time of treatment initiation, and in the latent reservoir (pre and post treatment).

## SIMULATION ALGORITHM

## Hybrid deterministic-stochastic method

The differential equation formulation of the model (Eq. (1)) can be easily numerically integrated but is unrealistic, as it results in all viral strains existing at low levels at all times due to continuous production of mutants, and therefore cannot accurately describe the waiting time until rare events occur nor the extinction of particular strains. However, a full stochastic simulation of realistic infection sizes ($\times 10^8$ infected cells or $\times 10^{10}$ virus) and strain structure (e.g. dozens of mutants) is computationally infeasible. To overcome these issues, we implemented a hybrid deterministic-stochastic approach to simulate the model.

We divided all the possible reactions of the system into those we assumed to take place in a continuous, deterministic fashion and those that occurred stochastically, based on whether they are likely to be rare or frequent transitions. Production and death of target cells, production and clearance of free virus, death of infected cells, and infection events that occur without mutation were simulated deterministically. Reactivation of latent cells and infection events in which mutation occurs were simulated stochastically. Stochastic events were simulated in a discrete-time manner, with time steps chosen as the minimum of the time between doses or 1 day. The number of cells of type $i$ exiting the latent reservoir in a time interval $\Delta t$ was drawn from $Poiss(A\, f_i\, \Delta t)$. The number of mutants produced by infection events was simply calculated by taking the number of new infections by each strain in the last time step (calculated from the numerical integration) and applying the mutation matrix **Q**.

## Initial conditions

We simulated maintenance therapy, in which participants have previously been on effective triple-drug antiretroviral therapy and are then switched to a simpler treatment regime (often fewer drugs) with the goal of maintaining a low viral load[64]. Therefore we initialized simulations with very low (<1 copies/ml) viral loads at levels sustained purely by release of virus from the latent reservoir (see above). Formally, we assumed that the total level of uninfected and infected cells, and free virus, were given by the steady state conditions of Equation (1) in the limit when $\beta = 0$:

$$
\begin{aligned}
x^* &= \frac{\lambda}{d_x} \\
y^* &= \frac{A}{d_y} \\
v^* &= \frac{Ak}{d_y c}
\end{aligned}
\tag{8}
$$

Note that with the parameters in Supplementary Table 3, this initial condition corresponds to a residual viral load around 0.1 copies/ml. Clinical studies with ultrasensitive assays find values of around 2 copies/ml [65], but this value represents all virions comprising the residual viremia whereas we only track the virions and reservoir cells that are replication-competent (since our estimates of reservoir exit rate were based on the timing of viral rebound [66], which must be caused by replication-competent virus). Differences in residual viremia values can be explained by the partially-defective nature of many provirus in the latent reservoir [67,68].

In both cases, the number of infected cells containing mutant viral strain $i$ was a random number drawn from a binomial distribution (approximated as Poisson-distributed) with an average given by the mutation-selection balance frequency $f_i$ (described above).

We assumed the first drug dose was taken and the post-dose peak concentration was $\Delta C$ (it will take multiple doses until steady state kinetics with peak level $C_{max}$ are reached).

**Declaring treatment failure**

Viral dynamics are tracked for a year-long period, and treatment failure is defined as two consecutive monthly measurements above 200 copies/ml. Treatment failure is classified as occurring due to resistance if over 20% of the viral population at failure contains resistance-associated mutations.

## Supplementary Methods

### Processing of drugs

There are several drugs approved for ART **(Supplementary Figure 4**), however, given the limitations on the size of a capsule that a patient can swallow, we could only accommodate drugs with a daily dose of <50 mg in our system. Based on this initial screening, we selected four drugs for our study. All drugs were purchased as weak acids or bases. As the commercial oral formulations contain salt forms of the drugs, we converted the parent drugs into their respective salt forms viz. dolutegravir sodium, cabotegravir sodium, rilpivirine hydrochloride and tenofovir alafenamide fumarate (TAF). Unless indicated otherwise, all experiments described in this manuscript were performed with the salt forms of the drugs.

TAF was synthesized as described before [1]. Briefly, tenofovir alafenamide (10g, 0.021 mol), fumaric acid (2.44 g, 0.021 mol), was suspended in acetonitrile (100mL) and heated to reflux with stirring until all solids dissolved. The reaction mixture was allowed to stand at room temperature to crystallize TAF, which was filtered, washed with acetonitrile (30mL), and dried before further use.

Dolutegravir sodium was synthesized as described by Johns *et al.*[2]. The parent acid (10g, 0.024 mol) was suspended in ethanol (30mL) and heated to reflux with stirring. Sodium hydroxide (8.4mL, 2N) was added to the solution while heating. The salt crystallized out on standing. It was filtered, washed with ethanol (50mL) and dried. Cabotegravir sodium was prepared similarly.

To prepare rilpivirine hydrochloride, the parent base (10g, 0.027 mol) was suspended in methanol (100mL) and heated to reflux with stirring. Isopropanol (50mL) containing 4mL of 6N HCl was added to the reaction mixture while heating. The salt was crystallized by placing the mixture at room temperature. The solid crystals were filtered and dried prior to use.

Nuclear magnetic resonance (NMR) and X-ray diffraction (XRD) analyses of DTG, RPV and CAB are shown in **Supplemental Figure 5** and **6** respectively.

### Estimating a plausible range for the impact of a weekly formulation on pre-exposure prophylaxis (PrEP) efficacy

To estimate a plausible effect size of a weekly oral option as an alternative to daily oral PrEP, we leveraged a meta-analysis of the difference in medication possession ratio (MPR), defined as the number of days of supply within the year that the patient has been prescribed, or proportion of days covered rate (PDR) for once-daily versus once-weekly oral dosing of any chronic medication for non-communicable diseases in non-trial settings [3]. The reported odds ratio of 1.90 (95% CI: 1.81-2.00) for MPR above 80% was translated to a mean difference (MD) of 15.5% (95% CI: 13.6-17.1%) using random effects meta-regression [4] of the studies investigated in [3].

Medication possession ratio is less meaningful in a clinical trial setting where medication refills are procedurally linked to follow-up visits and substantial effort is made to maximize trial retention. However, other measures of adherence, such as routine or unannounced pill counts, may still be variable among the retained population and provide insight into the relationship between adherence and plasma drug levels. We examined the correlation between pill count rate (PCR) and detectable levels of tenofovir, a component of oral PrEP, in the Partners PrEP study [5]. PCR had an 84% sensitivity to identify subjects with detectable tenofovir in plasma. We thus multiplied the estimated mean difference in adherence by 84% for a plausible mean difference in detectable drug levels between daily and weekly PrEP. It should be noted that PCR is a more conservative estimate of adherence (MPR≥PDR≥PCR) but these three estimates become more similar at high levels of

adherence (91% and 90% adherence by PCR in emtricitabine (FTC)/ TDF and TDF arms respectively in Partners PrEP).

Finally, the relationship between detectable drug levels and PrEP efficacy was estimated in a regression across clinical trials [6,7]. Strata of adherence within the trials were not considered to avoid the potential for confounding. A linear regression of PrEP efficacy versus the fraction of subjects with detectable plasma tenofovir in the VOICE, FEM-PrEP, iPrEx, iPrEx-OLE, Partners PrEP, and Partners Demonstration Studies [8] yielded a regression coefficient of 1.54% ($R^2$=0.94) increase in efficacy for a 1% increase in the fraction with detectable drug concentrations in plasma.

After combining these estimates, we arrived at plausible range of 20.1% (95% CI: 17.6–22.1%) increase in efficacy for weekly PrEP as compared to daily PrEP. However, because weekly PrEP will likely involve different drugs and may influence efficacy and coverage in unexpected ways, a wider range of efficacy and coverage assumptions were tested using a mathematical model.

**Structure, calibration and implementation of the epidemiological model**

The stochastic individual-based network model EMOD v2.5[9], calibrated to HIV epidemic trends in South Africa [10], was used to evaluate population-level impact of daily and weekly oral PrEP over time horizon of 20 years starting in 2017.

EMOD-HIV is an individual-based model that simulates transmission of HIV using an explicitly defined network of heterosexual relationships that are formed and dissolved according to age- and risk-dependent preference patterns[11]. Mother-to-child transmission is also included in the model. The modeled population was initiated in 1960 and projected forward with age-specific fertility rates and age/gender specific (non-AIDS) mortality rates obtained from UN World Population Prospects. The model was calibrated to match retrospective estimates of age-stratified, national-level prevalence, incidence, and ART coverage from four nationally representative HIV surveys in South Africa. The age patterns of sexual mixing were configured to match those observed in the rural, HIV-hyperendemic province of KwaZulu-Natal, South Africa[12]. Recently, a validation study showed that self-reported partner ages in this setting are relatively accurate, with 72% of self-reported estimates falling within two years of the partner's actual date of birth[13]. Further, the transmission patterns observed in EMOD are consistent with those revealed in a recent phylogenetic analysis of the age/gender patterns of HIV transmission in this setting[14].

Transmission rates within relationships were assumed to vary according to HIV disease stage, male circumcision, condom usage, co-infections, and antiretroviral therapy[15,16] with the latter reducing infectiousness by 92% within six months of treatment initiation-a conservative estimate based on observational data of serodiscordant couples in which outside partnerships could have contributed to HIV acquisition[17]. All scenarios included male medical circumcision[18] with an assumed 60% reduction in acquisition risk.

We configured the EMOD health care system module to follow trends in antiretroviral therapy (ART) expansion in South Africa. Diagnosis was assumed to be accessible via voluntary counselling and testing (VCT), antenatal and infant testing, symptom-driven testing, and low level of couples testing. The model includes loss to follow-up between diagnosis and staging, between staging and linkage to ART or pre-ART care, and during ART or pre-ART care[19,20], Two assumptions about future ART coverage were compared: one keeping present-day ART coverage levels of 60%, and another more optimistically assuming that the UNAIDS 90-90-90 targets will be achieved through expanded HIV testing, improved linkage to ART, and reduced lost-to-follow-up, such that in the model by 2032 90% of all individuals ever tested positive would be on ART.

Population-level impact of switching to weekly formulation for oral PrEP was assessed in terms of additional infections averted compared to daily PrEP. We assumed that a weekly formulation would increase efficacy from a baseline efficacy level of 50% by some positive increment, which was varied in sensitivity analysis. Efficacy was implemented as the reduction in the per-exposure probability of infection. In addition, we explored the possibility of an increase in coverage arising from greater acceptability of weekly oral PrEP, from baseline levels of 30% coverage to up to 60% coverage. PrEP was assumed to be available to high-risk populations, both genders, ages 15-29.

**Statistical analyses**

One-way ANOVA followed by a post-hoc Bonferroni test was used to compare flexural strength of Elastollan®R6000 to PCL and PLA, and to compare the Elastollan®R6000- Elastollan®1185 interface to other polymer interfaces. The effect of SGF on flexural strength of Elastollan®R6000 and Elastollan®R6000-Elastollan®1185 interface and differences in pharmacokinetics of sustained and immediate release dosage form was tested using two-sample t-test. Results of the funnel tests were evaluated using paired two-sample t-test. Variance in the groups being compared was generally similar. A p-value of less than 0.05 was considered statistically significant.

**Supplementary References**

1. Chapman, H. *et al.* PRACTICAL SYNTHESIS, SEPARATION, AND STEREOCHEMICAL ASSIGNMENT OF THE PMPA PRO-DRUG GS-7340. *Nucleosides, Nucleotides and Nucleic Acids* **20,** 621–628 (2001).

2. Johns, B. A., Duan, M. & Hakogi, T. Processes and intermediates for carbamoylpyridone hiv integrase inhibitors. (2010).

3. Iglay, K. *et al.* Systematic Literature Review and Meta-analysis of Medication Adherence With Once-weekly Versus Once-daily Therapy. *Clin. Ther.* **37,** 1813–21.e1 (2015).

4. Viechtbauer, W. Conducting Meta-Analyses in R with the metafor Package. *J. Stat. Softw.* **36,** (2010).

5. Donnell, D. *et al.* HIV Protective Efficacy and Correlates of Tenofovir Blood Concentrations in a Clinical Trial of PrEP for HIV Prevention. *JAIDS J. Acquir. Immune Defic. Syndr.* **66,** 340–348 (2014).

6. Fonner, V. A. *et al.* Effectiveness and safety of oral HIV preexposure prophylaxis for all populations. *AIDS* **30,** 1973–1983 (2016).

7. Bekker, L.-G. *et al.* Southern African guidelines on the safe use of pre-exposure prophylaxis in persons at risk of acquiring HIV-1 infection. *South. Afr. J. HIV Med.* **17,** 11 pages (2016).

8. Pre-Exposure Prophylaxis (PrEP) by the Numbers. *Global advocacy of HIV prevention* (2015). Available at: http://www.avac.org/sites/default/files/u3/By_The_Numbers_PrEP.pdf. (Accessed: 16th December 2016)

9. Source files for building the IDM EMOD disease transmission model. *Institute for Disease Modeling* (2016). Available at: https://github.com/InstituteforDiseaseModeling/EMOD. (Accessed: 1st January 2017)

10. O. Shisana, T. Rehle, L.C. Simbayi, K. Zuma, S. Jooste, N. Zungu, D. Labadarios, D. O. *South African National HIV Prevalence, Incidence and Behaviour Survey, 2012*. (HSRC Cape Town, 2014).

11. Bershteyn, A., Klein, D. J. & Eckhoff, P. A. Age-dependent partnering and the HIV transmission chain: a microsimulation analysis. *J. R. Soc. Interface* **10,** (2013).

12. Ott, M. Q., Bärnighausen, T., Tanser, F., Lurie, M. N. & Newell, M.-L. Age-gaps in sexual partnerships: seeing beyond 'sugar daddies'. *AIDS* **25,** (2011).

13. Harling, G., Tanser, F., Mutevedzi, T. & Bärnighausen, T. Assessing the validity of respondents' reports of their partners' ages in a rural South African population-based cohort. *BMJ Open* **5,** (2015).

14. de Oliveira, T. *et al.* Transmission networks and risk of HIV infection in KwaZulu-Natal, South Africa: a community-wide phylogenetic study. *Lancet HIV* **4,** e41–e50 (2017).

15. Attia, S., Egger, M., Müller, M., Zwahlen, M. & Low, N. Sexual transmission of HIV according to viral load and antiretroviral therapy: systematic review and meta-analysis. *AIDS* **23,** (2009).

16. Marconi, V. C. *et al.* Cumulative viral load and virologic decay patterns after antiretroviral therapy in HIV-infected subjects influence CD4 recovery and AIDS. *PLoS One* **6,** e17956 (2011).

17. O'Donnell, M. J. *et al.* Risk factors for ischaemic and intracerebral haemorrhagic stroke in 22 countries (the INTERSTROKE study): a case-control study. *Lancet* **376,** 112–123 (2017).

18. Connolly, C., Simbayi, L. C., Shanmugam, R. & Nqeketo, A. Male circumcision and its relationship to HIV infection in South Africa: Results of a national survey in 2002 . *SAMJ: South African Medical Journal* **98,** 789–794 (2008).

19. Mugglin, C. *et al.* Retention in Care of HIV-Infected Children from HIV Test to Start of

Antiretroviral Therapy: Systematic Review. *PLoS One* **8,** e56446 (2013).

20. Eaton, J. W. *et al.* Health benefits, costs, and cost-effectiveness of earlier eligibility for adult antiretroviral therapy and expanded treatment coverage: a combined analysis of 12 mathematical models. *Lancet Glob. Heal.* **2,** e23–e34 (2014).

21. Rosenbloom, D. I. S., Hill, A. L., Rabi, S. A., Siliciano, R. F. & Nowak, M. A. Antiretroviral dynamics determines HIV evolution and predicts therapy outcome. *Nat. Med.* **18,** 1378–85 (2012).

22. Wu, H. *et al.* Modeling long-term HIV dynamics and antiretroviral response: effects of drug potency, pharmacokinetics, adherence, and drug resistance. *J. Acquir. Immune Defic. Syndr.* **39,** 272–83 (2005).

23. Cadosch, D., Bonhoeffer, S. & Kouyos, R. Assessing the impact of adherence to anti-retroviral therapy on treatment failure and resistance evolution in HIV. *J. R. Soc. Interface* **9,** 2309–20 (2012).

24. Sanche, S. *et al.* A Mathematical Model to Predict HIV Virological Failure and Elucidate the Role of Lymph Node Drug Penetration. *CPT pharmacometrics Syst. Pharmacol.* (2017). doi:10.1002/psp4.12200

25. Perelson, A. S. & Ribeiro, R. M. Hepatitis B virus kinetics and mathematical modeling. *Semin. Liver Dis.* **24 Suppl 1,** 11–6 (2004).

26. Ke, R., Loverdo, C., Qi, H., Sun, R. & Lloyd-Smith, J. O. Rational Design and Adaptive Management of Combination Therapies for Hepatitis C Virus Infection. *PLoS Comput. Biol.* **11,** e1004040 (2015).

27. Rong, L. & Perelson, A. S. Mathematical analysis of multiscale models for hepatitis C virus dynamics under therapy with direct-acting antiviral agents. *Math. Biosci.* **245,** 22–30 (2013).

28. Schiffer, J. T. *et al.* Mathematical modeling of herpes simplex virus-2 suppression with pritelivir predicts trial outcomes. *Sci. Transl. Med.* **8,** 324ra15 (2016).

29. Ankomah, P., Johnson, P. J. T. & Levin, B. R. The pharmaco -, population and evolutionary dynamics of multi-drug therapy: experiments with S. aureus and E. coli and computer simulations. *PLoS Pathog.* **9,** e1003300 (2013).

30. Cadosch, D., Abel Zur Wiesch, P., Kouyos, R. & Bonhoeffer, S. The Role of Adherence and Retreatment in De Novo Emergence of MDR-TB. *PLoS Comput. Biol.* **12,** e1004749 (2016).

31. Nowak, M. A. & May, R. M. C. *Virus dynamics: mathematical principles of immunology and virology*. (Oxford University Press, USA, 2000).

32. Doitsh, G. *et al.* Abortive HIV Infection Mediates CD4 T Cell Depletion and Inflammation in Human Lymphoid Tissue. *Cell* **143,** 789–801 (2010).

33. Doitsh, G. *et al.* Cell death by pyroptosis drives CD4 T-cell depletion in HIV-1 infection. *Nature* **505,** 509–514 (2014).

34. Matrajt, L., Younan, P. M., Kiem, H.-P. & Schiffer, J. T. The Majority of CD4+ T-Cell Depletion during Acute Simian-Human Immunodeficiency Virus SHIV89.6P Infection Occurs in Uninfected Cells. *J. Virol.* **88,** 3202–3212 (2014).

35. Fauci, A. S. & Desrosiers, R. C. in *Retroviruses* (eds. Coffin, J. M., Hughes, S. H. & Varmus, H. E.) (Cold Spring Harbor Laboratory Press, 1997).

36. Coffin, J. & Swanstrom, R. HIV Pathogenesis: Dynamics and Genetics of Viral Populations and Infected Cells. *Cold Spring Harb. Perspect. Med.* **3,** (2013).

37. Anderson, R. W., Ascher, M. S. & Sheppard, H. W. Direct HIV Cytopathicity Cannot Account for CD4 Decline in AIDS in the Presence of Homeostasis: A Worst-Case Dynamic Analysis. *J. Acquir. Immune Defic. Syndr.* **17,** 245–252 (1998).

38. Douek, D. C. *et al.* HIV preferentially infects HIV-specific CD4+ T cells. *Nature* **417,** 95–98 (2002).

39. Arts, E. J. & Hazuda, D. J. HIV-1 Antiretroviral Drug Therapy. *Cold Spring Harb. Perspect. Med.* **2,** (2012).

40. Eisele, E. & Siliciano, R. F. Redefining the viral reservoirs that prevent HIV-1 eradication. *Immunity* **37,** 377–388 (2012).

41. Davey, Jr., R. T. *et al.* HIV-1 and T cell dynamics after interruption of highly active antiretroviral therapy (HAART) in patients with a history of sustained viral suppression. *Proc. Natl. Acad. Sci. USA* **96,** 15109–15114 (1999).

42. Ruiz, L. *et al.* Structured treatment interruption in chronically HIV-1 infected patients after long-term viral suppression. *AIDS* **14,** 397 (2000).

43. Archin, N. M. *et al.* Immediate antiviral therapy appears to restrict resting CD4+ cell HIV-1 infection without accelerating the decay of latent infection. *Proc. Natl. Acad. Sci. U. S. A.* **109,** 9523–9528 (2012).

44. Siliciano, J. D. *et al.* Long-term follow-up studies confirm the stability of the latent

reservoir for HIV-1 in resting CD4+ T cells. *Nat Med* **9,** 727–728 (2003).

45. Chou, T.-C. Derivation and properties of Michaelis-Menten type and Hill type equations for reference ligands. *J. Theor. Biol.* **59,** 253–276 (1976).

46. Shen, L. *et al.* Dose-response curve slope sets class-specific limits on inhibitory potential of anti-HIV drugs. *Nat. Med.* **14,** 762–766 (2008).

47. Jilek, B. L. *et al.* A quantitative basis for antiretroviral therapy for HIV-1 infection. *Nat. Med.* **18,** 446–451 (2012).

48. Margolis, D. A. & Boffito, M. Long-acting antiviral agents for HIV treatment. *Curr. Opin. HIV AIDS* **10,** 246–52 (2015).

49. Landovitz, R. J., Kofron, R. & McCauley, M. The promise and pitfalls of long-acting injectable agents for HIV prevention. *Curr. Opin. HIV AIDS* **11,** 122–8 (2016).

50. Kirtane, A. R., Langer, R. & Traverso, G. Past, Present, and Future Drug Delivery Systems for Antiretrovirals. *J. Pharm. Sci.* **105,** 3471–3482 (2016).

51. Glaubius, R. L. *et al.* Deciphering the Effects of Injectable Pre-exposure Prophylaxis for Combination Human Immunodeficiency Virus Prevention. *Open Forum Infect. Dis.* **3,** ofw125 (2016).

52. Swanstrom, R. & Coffin, J. HIV-1 pathogenesis: the virus. *Cold Spring Harb. Perspect. Med.* **2,** a007443 (2012).

53. Josefsson, L. *et al.* Single cell analysis of lymph node tissue from HIV-1 infected patients reveals that the majority of CD4+ T-cells contain one HIV-1 DNA molecule. *PLoS Pathog.* **9,** e1003432 (2013).

54. Haase, A. T. Population Biology of HIV-1 Infection: Viral and CD4+ T Cell Demographics and Dynamics in Lymphatic Tissues. *Annu. Rev. Immunol.* **17,** 625–656 (1999).

55. Sampah, M. E. S., Shen, L., Jilek, B. L. & Siliciano, R. F. Dose-response curve slope is a missing dimension in the analysis of HIV-1 drug resistance. *Proc. Natl. Acad. Sci. U. S. A.* **108,** 7613–7618 (2011).

56. Gray, W. T. *et al.* Potent Inhibitors Active against HIV Reverse Transcriptase with K101P, a Mutation Conferring Rilpivirine Resistance. *ACS Med. Chem. Lett.* **6,** 1075–9 (2015).

57. Laskey, S. B. & Siliciano, R. F. Quantitative evaluation of the antiretroviral efficacy of

dolutegravir. *JCI insight* **1,** e90033 (2016).

58. AIDS info Drug Database. *Department of Health and Human Services, US* (2017). Available at: https://aidsinfo.nih.gov/drugs. (Accessed: 1st January 2017)

59. Bangsberg, D. R. *et al.* Adherence-resistance relationships for protease and non-nucleoside reverse transcriptase inhibitors explained by virological fitness. *AIDS* **20,** 223–31 (2006).

60. Mansky, L. M. & Temin, H. M. Lower in vivo mutation rate of human immunodeficiency virus type 1 than that predicted from the fidelity of purified reverse transcriptase. *J. Virol.* **69,** 5087–5094 (1995).

61. Abram, M. E., Ferris, A. L., Shao, W., Alvord, W. G. & Hughes, S. H. Nature, Position, and Frequency of Mutations Made in a Single Cycle of HIV-1 Replication. *J. Virol.* **84,** 9864–9878 (2010).

62. *HIV Sequence Compendium 2010*. (Theoretical Biology and Biophysics Group, Los Alamos National Laboratory, NM, 2010).

63. Crow, J. F. & Kimura, M. *An introduction to population genetics theory*. (Burgess Pub. Co., 1970).

64. Havlir, D. V *et al.* Maintenance antiretroviral therapies in HIV infected patients with undetectable plasma HIV RNA after triple-drug therapy. AIDS Clinical Trials Group Study 343 Team. *N. Engl. J. Med.* **339,** 1261–1268 (1998).

65. Dinoso, J. B. *et al.* Treatment intensification does not reduce residual HIV-1 viremia in patients on highly active antiretroviral therapy. *Proc. Natl. Acad. Sci.* **106,** 9403 (2009).

66. Hill, A. L., Rosenbloom, D. I. S., Fu, F., Nowak, M. A. & Siliciano, R. F. Predicting the outcomes of treatment to eradicate the latent reservoir for HIV-1. *Proc. Natl. Acad. Sci.* **111,** 13475–13480 (2014).

67. Ho, Y.-C. *et al.* Replication-Competent Noninduced Proviruses in the Latent Reservoir Increase Barrier to HIV-1 Cure. *Cell* **155,** 540–551 (2013).

68. Bruner, K. M. *et al.* Defective proviruses rapidly accumulate during acute HIV-1 infection. *Nat. Med.* **22,** 1043–1049 (2016).

69. Guyton, A. C. & Hall, J. E. in *Textbook of Medical Physiology* (Saunders Co., 2005).

70. Zhang, Z.-Q. *et al.* Kinetics of CD4+ T cell repopulation of lymphoid tissues after treatment of HIV-1 infection. *Proc. Natl. Acad. Sci. U. S. A.* **95,** 1154–1159 (1998).

71. Ribeiro, R. M. *et al.* Estimation of the Initial Viral Growth Rate and Basic Reproductive Number during Acute HIV-1 Infection. *J. Virol.* **84,** 6096–6102 (2010).

72. Macallan, D. C. *et al.* Rapid Turnover of Effector–Memory CD4+ T Cells in Healthy Humans. *J. Exp. Med.* **200,** 255–260 (2004).

73. Markowitz, M. *et al.* A Novel Antiviral Intervention Results in More Accurate Assessment of Human Immunodeficiency Virus Type 1 Replication Dynamics and T-Cell Decay In Vivo. *J. Virol.* **77,** 5037–5038 (2003).

74. De Boer, R. J., Ribeiro, R. M. & Perelson, A. S. Current Estimates for HIV-1 Production Imply Rapid Viral Clearance in Lymphoid Tissues. *PLoS Comput Biol* **6,** e1000906 (2010).

75. Ramratnam, B. *et al.* Rapid production and clearance of HIV-1 and hepatitis C virus assessed by large volume plasma apheresis. *Lancet* **354,** 1782–1785 (1999).

76. Wainberg, M. A. & Han, Y.-S. Will drug resistance against dolutegravir in initial therapy ever occur? *Front. Pharmacol.* **6,** 90 (2015).

77. Nakahara, K. *et al.* Secondary mutations in viruses resistant to HIV-1 integrase inhibitors that restore viral infectivity and replication kinetics. *Antiviral Res.* **81,** 141–146 (2009).

78. Seki, T. *et al.* Effects of raltegravir or elvitegravir resistance signature mutations on the barrier to dolutegravir resistance in vitro. *Antimicrob. Agents Chemother.* **59,** 2596–606 (2015).

79. Anstett, K., Cutillas, V., Fusco, R., Mesplède, T. & Wainberg, M. A. Polymorphic substitution E157Q in HIV-1 integrase increases R263K-mediated dolutegravir resistance and decreases DNA binding activity. *J. Antimicrob. Chemother.* **71,** 2083–8 (2016).

80. Kobayashi, M. *et al.* In Vitro antiretroviral properties of S/GSK1349572, a next-generation HIV integrase inhibitor. *Antimicrob. Agents Chemother.* **55,** 813–21 (2011).

81. Xu, H.-T. *et al.* Role of the K101E substitution in HIV-1 reverse transcriptase in resistance to rilpivirine and other nonnucleoside reverse transcriptase inhibitors. *Antimicrob. Agents Chemother.* **57,** 5649–57 (2013).

82. Wang, J. *et al.* The Non-nucleoside Reverse Transcriptase Inhibitor Efavirenz Stimulates Replication of Human Immunodeficiency Virus Type 1 Harboring Certain Non-nucleoside Resistance Mutations. *Virology* **402,** 228–237 (2010).

83. Xu, H.-T. *et al.* Compensation by the E138K Mutation in HIV-1 Reverse Transcriptase for

Deficits in Viral Replication Capacity and Enzyme Processivity Associated with the M184I/V Mutations. *J. Virol.* **85,** 11300–11308 (2011).

84. Picchio, G. R. *et al.* Prevalence in the USA of rilpivirine resistance-associated mutations in clinical samples and effects on phenotypic susceptibility to rilpivirine and etravirine. *Antivir. Ther.* **19,** 819–23 (2014).

85. Rimsky, L. *et al.* Genotypic and phenotypic characterization of HIV-1 isolates obtained from patients on rilpivirine therapy experiencing virologic failure in the phase 3 ECHO and THRIVE studies: 48-week analysis. *J. Acquir. Immune Defic. Syndr.* **59,** 39–46 (2012).

86. Haddad, M. Mutation Y188L of HIV-1 Reverse Transcriptase is Strongly Associated with Reduced Susceptibility to Rilpivirine. in (2012).

87. Azijn, H. *et al.* TMC278, a next-generation nonnucleoside reverse transcriptase inhibitor (NNRTI), active against wild-type and NNRTI-resistant HIV-1. *Antimicrob. Agents Chemother.* **54,** 718–27 (2010).
